# Acute Effects of Dietary Fiber in Starchy Foods on Glycemic and Insulinemic Responses: A Systematic Review of Randomized Controlled Crossover Trials

**DOI:** 10.3390/nu15102383

**Published:** 2023-05-19

**Authors:** Sofia Tsitsou, Christina Athanasaki, George Dimitriadis, Emilia Papakonstantinou

**Affiliations:** 1 Laboratory of Dietetics and Quality of Life, Department of Food Science and Human Nutrition, School of Food and Nutritional Sciences, Agricultural University of Athens, 75 Iera Odos, 11855 Athens, Greece; stsitsou@aua.gr (S.T.); chrathanasaki@gmail.com (C.A.); 2 2nd Department of Internal Medicine, Research Institute and Diabetes Center, National and Kapodistrian University of Athens, Attikon University Hospital, 1 Rimini Street, 12462 Haidari, Greece; gdimitr@med.uoa.gr

**Keywords:** dietary fiber, soluble dietary fiber, insoluble dietary fiber, resistant starch, starchy foods, glycemic responses, insulinemic responses, humans

## Abstract

Dietary fiber (DF) consumption has been associated with improved glycemic control in epidemiological and long-term interventional studies. However, its acute effects are not yet clear. This systematic review aims to elucidate the postprandial effects of DF in starchy products on glycemia and insulinemia. An electronic search of databases was conducted, and forty-one records met the inclusion criteria and underwent a risk-of-bias assessment. It was shown that soluble DF does not clearly affect glycemia in individuals with normal weight, while resistant starch may be more effective in flattening glycemic responses. Concerning insulinemia, both soluble DF and resistant starch have mixed results, with either favorable or no effects. Data on insoluble DF and glucose metabolism are scarce. The same mixed results for glycemia can be seen in healthy volunteers with overweight/obesity, while resistant starch seems to improve insulinemic responses. Finally, more studies need to examine the acute effects of DF in starchy foods on glucose metabolism and insulin secretion in individuals facing glucose abnormalities. Additionally, more studies are needed to prove whether ingesting high-fiber carbohydrate-containing products per se can result in blunted glycemic and insulinemic responses and which DF type and amount are more effective.

## 1. Introduction

Improving diet and lifestyle is considered the keystone for the prevention and treatment of obesity and glucose metabolism disorders such as type 2 diabetes mellitus (T2DM) [1]. These chronic diseases are spreading rapidly, and poor nutrition is the main contributing to their epidemic status [2]. As reported by several nutritional guidelines, dietary fiber constitutes a key component of a healthy diet [3]. The definition of dietary fiber has changed over the years. Dietary fiber was first defined as the part of food derived from the cellular walls of plants that are poorly digested by humans [4]. In 1976, Trowell et al. redefined dietary fiber as edible plant polysaccharides, such as gums, mucilage, hemicellulose, pectic substances, and a non-carbohydrate component, lignin, naturally occurring in foods [5]. Nowadays, the European Food Safety Authority (EFSA) defines dietary fiber as non-digestible carbohydrates plus lignin, which is resistant to hydrolysis by human digestive enzymes [4]. This term was modified to specify oligosaccharides, including inulin and resistant starches [6].

Dietary fiber is a chemically heterogeneous group of compounds with variable molecular sizes and different physicochemical properties [7]. Several classification systems have been suggested for the components of dietary fiber based on several parameters, e.g., gastrointestinal solubility, role in the plant, and site of digestion; however, none seems to be universally accepted [8]. The traditional classification of dietary fiber is often based on its water solubility, viscosity, and fermentability [9]. Although solubility per se is an essential determinant of physiological responses, fermentability and viscosity are prone to play a more pronounced role in the physiological benefits to human health [10]. Water-soluble or, simply, soluble dietary fiber, including pectin, mucilage, gums, fructans, β-glucan, and some resistant starches, is fermented in the colon, affecting nutrient absorption in the small intestine; some fruits, vegetables, oats, and barley contain this type of fiber [7,9,11]. In contrast, insoluble dietary fiber, chiefly including lignin, cellulose, and hemicellulose, contributes to quick gastric emptying and may only be fermented to a limited extent in the colon, thus promoting digestive regularity; wholegrain products, bran, nuts, and seeds are rich in this fiber type [7,9,11,12]. However, foods may contain different types of dietary fiber with contradicting actions.

According to the Academy of Nutrition and Dietetics, individuals should consume an adequate daily amount of dietary fiber, which is equal to 14 g of total fiber per 1000 kcal, or 25 g/day for adult women and 38 g/day for adult men. These guidelines are based on the evident protection provided by dietary fiber against coronary heart disease [13], and they have also been adopted by the American Diabetes Association for the prevention and treatment of prediabetes and T2DM [14]. In Europe, a product must contain at least 3 g of fiber per 100 g of a product or at least 1.5 g of fiber per 100 kcal to qualify for a “source of fiber” claim. To be a “high-fiber” food, the product must contain at least 6.0 g of fiber per 100 g of a product or at least 3.0 g of fiber per 100 kcal [15].

Dietary fiber has been extensively studied in the last few decades for its physiological health benefits [16]. Dietary fiber possesses all the properties required to be considered a crucial ingredient in the formulation of functional foods due to its beneficial health effects [17]. Evidence from epidemiological and experimental studies has associated the consumption of dietary fiber with the decreased incidence of a wide range of diseases, such as obesity, colon cancer, and heart disease [17,18,19]. Specifically, dietary fiber may add volume to the diet and, as a result, may reduce appetite by making the individual feel full faster. Moreover, its consumption (1) may lower total and low-density lipoprotein (LDL) cholesterol levels, (2) may regulate blood pressure, (3) may add bulk to the stool, protecting against constipation, (4) may speed up the passage of food through the digestive system, facilitating regularity, and (5) may balance intestinal pH and stimulate intestinal fermentation and the production of short-chain fatty acids [17,20].

As the human body is unable to absorb and break down fiber, it is widely believed that dietary fiber, and more specifically soluble dietary fiber, does not cause a spike in blood glucose the way that other carbohydrates can [21,22]. When soluble dietary fiber interacts with water, it forms a gel. As a result of this viscous solution, the emptying of the stomach, the passage of food through the digestion tract, and the absorption of glucose are all slowed. The gradual absorption of ingested glucose may enhance insulin economy and glucose disposal, prevent late postprandial hypoglycemia, ameliorate glucose fluctuations, and increase tissue sensitivity to insulin; these effects are most important in individuals facing glucose abnormalities, such as prediabetes or T2DM [22,23]. The rates of gastric emptying and intestinal glucose absorption play a decisive role in the magnitude of postprandial hyperglycemia and hyperinsulinemia [24]. Slower rates of glucose delivery into the small intestine and the gradual absorption of ingested glucose enhance insulin economy and glucose disposal and improve insulin sensitivity [24]. It is believed that dietary fiber from cereal may be more effective in improving body weight and insulin sensitivity than that from fruits and vegetables [11,25]. Epidemiological studies have also linked high-fiber intake to a reduced risk of T2DM [26,27]. A recent systematic review and meta-analysis of twenty-one randomized controlled trials (RCTs) in patients with T2DM reported that, compared to controls, dietary fiber at a median daily dose of 10 g/day for a mean intervention duration of 8 weeks significantly reduced glycated hemoglobin A1c (HbA1c), fasting glucose and insulin, and Homeostatic Model Assessment for Insulin Resistance (HOMA-IR) [28]. Both soluble fiber products and fiber from natural foods were found to be effective in improving glycemic control and insulin sensitivity in T2DM patients, with the former yielding better effects [28]. Concerning the type of fiber that is more effective in ameliorating postprandial glycemic and insulinemic responses, the evidence is not consistent. On the one hand, it is believed that diets high in insoluble and only moderately fermentable cereal fiber reduce the risk of developing T2DM [29], whereas, in some other studies, naturally available high-fiber products, such as oats and barley, containing soluble β-glucan have been connected with improved glycemic control [11,16]. Finally, dietary fiber is thought to reduce the glycemic index (GI) of carbohydrate-containing products [30]. The GI is an international standardized index that describes the postprandial increase in blood glucose. The FAO/WHO recommends a low-GI diet to prevent diseases, such as obesity, heart disease, and T2DM [30].

There is not yet a well-established and consistent opinion on the effects of dietary fiber on glucose metabolism. To our knowledge, there are no systematic reviews evaluating the acute effects of dietary fiber incorporated in starchy products on glycemic and insulinemic responses in both healthy individuals and patients with various health statuses. It is crucial to understand whether the ingestion of carbohydrate-containing products that differ in the type and/or amount of dietary fiber results in differentiated glycemic indices at an acute level.

## 2. Materials and Methods

The review protocol was registered with and published on PROSPERO (registration number: CRD42023386849). This systematic review was organized according to the guidelines of the Preferred Reporting Items for Systematic Reviews and Meta-Analyses (PRISMA) statements [31].

### 2.1. Search Strategy

**Figure 1:**
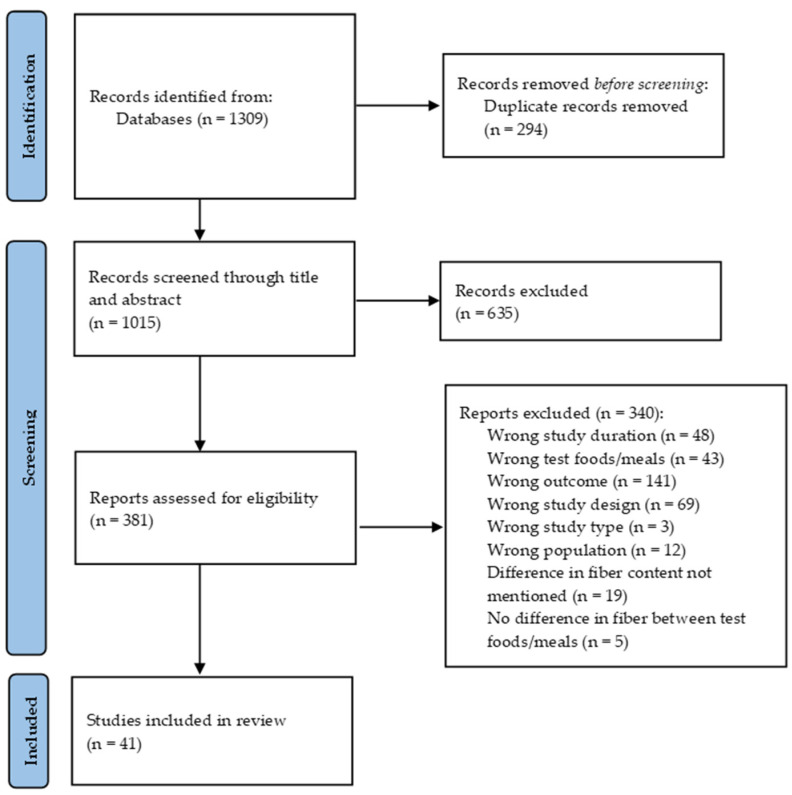
PRISMA flow diagram of included studies.

Two reviewers (S.T. and C.A.) conducted separate systematic searches of PUBMED, Google Scholar, and SCOPUS databases for eligible studies. The research started in January 2023 and finished in February 2023. The last day of searching was the 26th of February 2023. Specific language and age criteria were applied. The medical subject headings (MeSH terms) and keywords chosen were the following: (“dietary fiber” OR “dietary fibre” OR “soluble dietary fiber” OR “insoluble dietary fiber” OR “resistant starch” OR “pectin” OR “β-glucan” OR “psyllium” OR “guar gum” OR “wholegrain” OR “wheat” OR “oat(s)” OR “barley” OR “rye” OR “carob” OR “corn” OR “durum” OR “seed(s)” OR “chia seed(s)” OR “flaxseed” OR “bread” OR “pasta” OR “spaghetti” OR “all-bran” OR “soy” OR “pulses” OR “legume(s)” OR “chickpeas” OR “bean(s)” OR “lentil(s)” OR “enriched/enhanced/fortified with fiber”) AND (“blood glucose” OR “postprandial glucose” OR “postprandial insulin” OR “glyc(a)emic responses” OR “insulin(a)emic responses” OR “glyc(a)emia” OR “insulin(a)emia” OR “glucose metabolism” OR “glyc(a)emic variability” OR “glyc(a)emic excursions” OR “glycemic index” OR “area under the curve” OR “glycemic load” OR “insulin resistance” OR “insulin sensitivity” OR “continuous glucose monitoring”). The results of the searches are represented in Figure 1 (PRISMA flow diagram).

### 2.2. Eligibility Criteria

**Figure 2:**
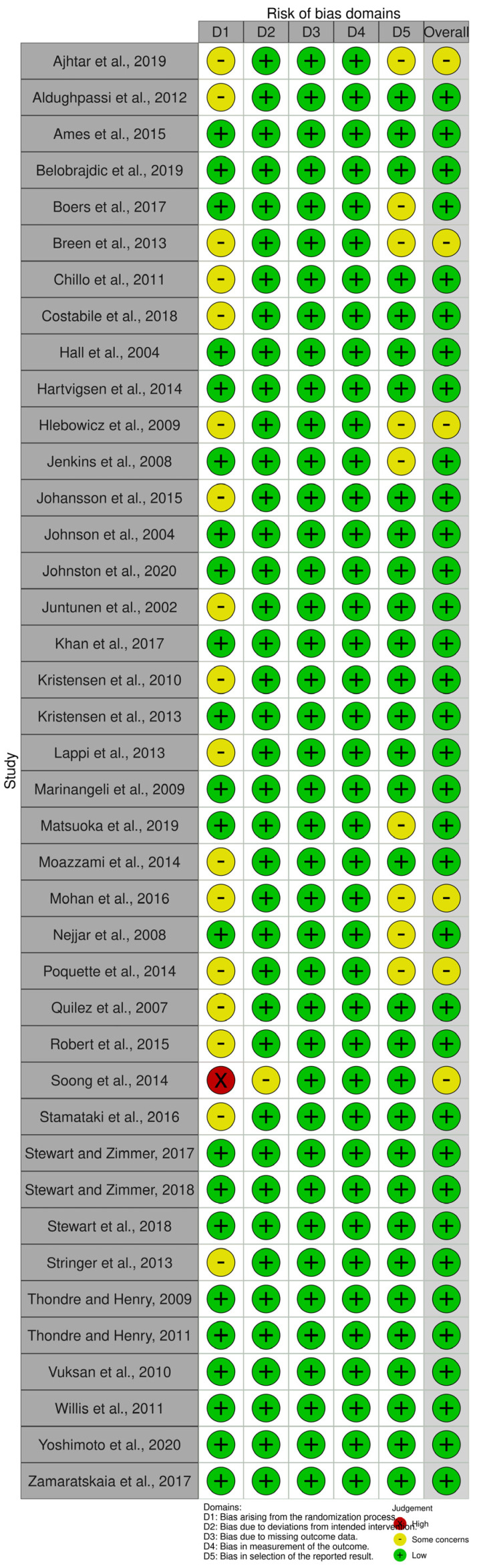
Domains of risk of bias [32,33,34,35,36,37,38,39,40,41,42,43,44,45,46,47,48,49,50,51,52,53,54,55,56,57,58,59,60,61,62,63,64,65,66,67,68,69,70,71,72].

**Figure 3:**
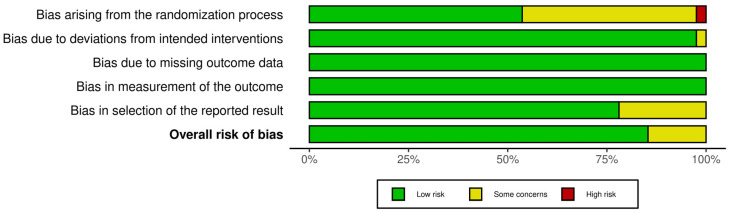
Overall risk of bias.

In this systematic review, studies examining the acute effects of dietary fiber existing in or added to starchy foods, e.g., bread, spaghetti, and biscuits, on glycemic and insulinemic responses were included. The inclusion criteria selected were the following: (1) population: adults aged 19+ years; (2) study design: RCTs (Figure 2); (3) language: studies in English only; (4) humans only; (5) publication year: no restriction on the year published; (6) full texts only; (7) studies evaluating different types, e.g., soluble and insoluble, and/or amounts of dietary fiber existing naturally in or added to starchy products, e.g., bread and spaghetti; these products could be examined alone or as a part of a specific meal; and (8) primary outcomes: differences in postprandial glycemic and insulinemic responses between different test meals (acute effects). The exclusion criteria selected were the following: (1) population: studies in children and adolescents; (2) study design: non-randomized clinical trials, feasibility studies, prospective studies, commentaries/letters, editorials, systematic reviews, narrative reviews, reviews, and meta-analyses; (3) studies conducted in animals; (4) studies examining the long-term (more than a few hours) effects of dietary fiber on glucose metabolism and insulin sensitivity; (5) studies on foods other than starchy foods, e.g., beverages or fruit juices differing in dietary fiber; (6) studies in which the examined foods did not differ in their dietary fiber content or in which this content was not mentioned; (7) studies examining whole diets or dietary patterns containing different amounts and/or types of dietary fiber; and (8) studies in individuals with type 1 diabetes mellitus, gestational diabetes mellitus, or inflammatory or kidney diseases.

### 2.3. Selection of Studies and Data Extraction

All research results were imported into a citation manager, and all duplicate records were automatically removed. The next step was screening titles and abstracts retrieved through the search strategy and identifying studies meeting the inclusion criteria. Then, the full texts of the identified studies were retrieved and assessed for eligibility. Finally, any disagreement between reviewers was resolved through a discussion with a third author (E.P.).

The following information was obtained from the selected studies: author names; year of publication; country in which it was conducted; study design and duration; the health status of the participants and characteristics concerning age, sex, and body mass index; sample size; types and amounts of the dietary fiber tested; macronutrient analysis of the test meals; and outcomes for glycemia and/or insulinemia.

### 2.4. Risk of Bias

The revised Cochrane risk-of-bias tool for randomized trials (RoB 2) was used to assess the bias of the selected randomized controlled trials with a crossover design. This tool describes five main domains of bias: randomization process, deviations from intended interventions, missing outcome data, measurement of the outcome, and selection of reported results [73]. The robvis visualization tool was used to create all the relative plots for the studies included in the final analysis (Figure 2 and Figure 3) [74].

## 3. Results

The search strategy in the databases revealed 1309 records in total (Figure 1). After removing 294 duplicates, 1015 records were identified for screening through titles and abstracts. Of these, 635 references did not meet the inclusion criteria, while 380 articles underwent full-text review. Further searching of the reference lists of the reviewed papers resulted in 1 more article. Of the 381 records assessed for eligibility, 48 were excluded due to the wrong study duration (long-term studies), and 3 and 69 were not the appropriate study type and study design, respectively. Moreover, 141 articles did not have a relevant outcome, 43 did not contain the appropriate test foods/meals (other than starchy foods), and 19 did not mention differences in fiber content between test meals, while in 5 papers, the test meals/foods had similar fiber content, so its effects could not be evaluated. In the final analysis, 41 eligible studies were included.

### 3.1. Study Characteristics

This systematic review included 41 RCTs with a crossover design, blinded or not (Table 1). All participants were adults, and the sample sizes of the studies ranged from 8 individuals [47] to 50 [59]. Consequently, all studies are characterized by small but adequate sample sizes, according to a power analysis and the studies’ designs. Glycemic and insulinemic responses were evaluated for 90 [50,51] to 420 min [37]. However, in most of the studies, the postprandial effects of the test foods/meals on glucose and insulin levels were assessed for either 120 [33,34,38,40,41,46,47,48,56,57,60,64,65,68,70,71] or 180 min [32,36,39,44,45,51,52,55,59,61,63,67,69]. Finally, all the studies were conducted between 2002 [44] and 2022 [72].

From the forty-one RCTs included in the analysis, 41.5% were conducted in Europe, mainly in Scandinavia (Sweden, Finland, and Denmark) [33,35,36,37,44,47,48,50,53,54,55,58,63,64,66,72], and 31.7% were performed in North America, with the majority of the studies originating from Canada [32,38,39,40,41,42,45,51,52,56,67,69,70]. The remaining 17% were from Asia [34,46,49,57,59,60,62], and 9.8% were from Australia [61,65,68,71]. Both males and females participated in almost all studies, except for two trials that included only male participants [37,69] and one trial involving only females [63]. Moreover, most of the studies evaluated the acute effects of dietary fiber on glucose metabolism in healthy participants. Three studies tested individuals with T2DM [35,51,52], while one study included subjects with metabolic syndrome [53], and another study involved healthy subjects with self-reported gastrointestinal symptoms after the ingestion of cereal foods, particularly rye bread [66].

The starchy foods tested in the included studies, either alone or as part of a meal, were different types of breads [35,36,44,50,53,56,60,61,63,66,68,69,71], crispbreads [54,58], chapattis [34,47,48], flatbreads [46,59], pasta or spaghetti [33,36,42,43,72], noodles [62], rice [49], biscuits or cookies [41,42,51,65], scones [39], tortillas [32], crackers [52], buns [37,46], muffins [40,45,57,64,67], or other snacks made with different flours [70]. All these foods differed mainly in the flour used for their preparation. The flours that were examined are the following: wholegrain or whole-meal or whole-wheat or whole-kernel or refined wheat, rye, barley, sorghum, or semolina, durum or not, with or without sourdough, and flours from legumes, pulses, vegetables, or seeds, e.g., fenugreek and chia. Finally, in eleven out of forty-one studies, the starchy foods were tested as part of a meal [34,36,37,43,44,58,60,63,66,68,70].

The majority of the included studies evaluated the glycemic and/or insulinemic responses to the soluble dietary fiber β-glucan [33,38,44,45,47,48,50,53,57,59,60,66,69]. Some other types of soluble dietary fiber that were tested are the following: arabinoxylan [36,50,53,54,58,66], galactomannan [46], glucomannan [51], inulin [55], pectin [45], guar gum [45,64], fructan [66], xanthan [51], and cellulose [64]. The second most studied dietary fiber is resistant starch [39,40,41,49,61,67]. Finally, some studies examined the acute effects of mixed fiber (soluble, insoluble, and resistant starch) on glycemia and/or insulinemia [32,42,54,58,63,64,70,71]. All studies compared two or more starchy foods or meals differing in the total dietary fiber content. This ranged from 0 g (white bread as a control test food/meal) [50] to 26.87 g (tortilla made with bran flour and high insoluble dietary fiber and medium β-glucan contents) [32].

The assessment of the risk of bias was performed separately for each study. Thirty-five out of the forty-one studies had a low overall risk of bias, while the remaining six studies had some concerns due to bias arising from the randomization process, due to deviations from the intended intervention, and due to bias in the selection of the reported result (Figure 2 and Figure 3). In total, the main issues pertaining to the selected records concerned missing details of the randomization process and, specifically, missing data on the blinding of the participants and/or researchers, as well as on the selection of a prespecified analysis.

### 3.2. Main Exposures

The main outcomes of this systematic review were changes in glycemic and insulinemic responses a few hours, e.g., 120 min (acute), after the consumption of starchy foods e.g., bread or meals containing starchy foods, differing in the amount and/or type of dietary fiber. The indices measured after the analysis were the following: postprandial glucose (PPG), postprandial insulin (PPI), glycemic index (GI), glycemic load (GL), insulinemic index (II), area under the curve (AUC) for glucose and insulin, incremental AUC (iAUC), peak glucose and insulin or maximal concentrations (Cmax), and peak time for glucose and insulin.

### 3.3. Effects of Dietary Fiber on Glycemic Responses

All forty-one included studies assessed the acute effects of various types of dietary fiber on glycemic responses not only in healthy participants [32,33,34,36,37,38,39,40,41,42,43,44,45,46,47,48,49,50,51,52,54,55,56,57,58,59,60,61,62,63,64,65,67,68,69,70,71,72] but also in individuals with different health conditions [35,51,52,53,66].

#### 3.3.1. Healthy Individuals

##### Normal Weight

Most of the studies conducted in healthy volunteers with normal weight assessed the acute effects of soluble dietary fiber, e.g., β-glucan and guar gum, in starchy foods on glycemia [33,36,37,44,45,46,47,48,50,51,54,55,57,58,59,60,72]. Concerning the glucose iAUC, four RCTs showed a reduction after the ingestion of starchy products with soluble fiber [33,46,47,59], while in five trials, no differences were observed between test meals [37,48,50,55,72], and in one study only, the iAUC increased dose-dependently with the fiber content of muffins [45]. In the study conducted by Kristensen et al. (2010), it was observed that wholegrain bread resulted in a higher glucose iAUC compared to wholegrain pasta, despite its higher content in total dietary fiber (11.0 vs. 5.0 g) [36]. Glycemic responses and PPG did not differ between the starchy products tested in five of the studies included in this systematic review [36,37,50,54,58]. In contrast to these observations, two RCTs showed improved glycemic responses after the consumption of biscuits and chapattis high in soluble fiber in healthy participants [47,51], while in the trial by Juntunen et al., the consumption of β-glucan rye bread (17.1 g fiber) and whole-meal pasta made with dark durum wheat (5.6 g fiber) led to worse glycemic responses in comparison with wheat white bread (3.1 g of fiber) [44]. In the study by Papakonstantinou et al. in healthy subjects, it was observed that wholegrain spaghetti made with wholegrain hard wheat flour (7.0 g fiber) resulted in higher glycemic responses compared to glucose (reference food) [72]. Additionally, five studies reported lower peak glucose values after the consumption of soluble dietary fiber from starchy products (spaghetti, bread, and muffins) [44,45,57,60,72]. As regards resistant starch, six studies in total evaluated its acute effects on glycemia when incorporated in starchy foods [40,41,49,61,67,68]. Five of them resulted in a lower glucose iAUC of the tested products (muffins, rice, bread, and cookies) compared to the control food [40,41,49,61,67], and three studies reported decreased Cmax for glucose [40,41,61]. Moreover, although in the study by Poquette et al., there were no differences in glycemic responses after the ingestion of muffins made either with whole-wheat flour or wholegrain sorghum [67], in two other trials, a reduction in the glycemic response was reported after the consumption of bread and cookies high in resistant starch [41,68]. When Yoshimoto et al. tested the effects of insoluble dietary fiber in noodles, they found no differences in the glucose iAUC between the tested products [62]. Furthermore, of the studies in which the fiber type was not mentioned, two reported no difference in the glucose iAUC [43,65], while the remaining two studies resulted in a lower glucose iAUC after the consumption of chapattis (supplemented with vegetable or bean powder) and bread (different doses of chia seeds) compared to the control products [34,56]. Finally, three studies examined the combination of soluble and insoluble fiber in starchy products, which resulted either in a decreased iAUC [42] or in no difference in the glycemic responses between the test foods [70] or in a lower GI of bread made with lupin flour [71]. The incorporation of both soluble and insoluble dietary fiber as well as resistant starch in tortillas led to a reduced glucose iAUC as the fiber content increased [32].

In conclusion, it is not clear whether the addition of soluble dietary fiber to starchy products improves the acute glycemic responses in healthy individuals with normal weight. However, this is more evident with resistant starch, while data on insoluble fiber are scarce.

##### Overweight and Obesity

The short-term effects of dietary fiber in starchy foods on glycemia in healthy subjects with overweight/obesity were tested in five studies [38,39,63,64,69]. In two of the studies, the consumption of breads high in β-glucan resulted in lower glycemic responses [38,69]. In contrast, in the study conducted by Quilez et al., it was found that a low-calorie muffin containing 6.3% dietary fiber led to similar glycemic responses to that after consuming a plain muffin with 1.5% fiber but a reduced responses compared with white bread (2.7% fiber) [64]. The glycemic responses to the muffin under investigation was higher in overweight individuals compared to the normal group [64]. In another study by Stewart et al., the consumption of a scone with type-4 resistant starch (17.5 g fiber) resulted in a reduced glucose Cmax and glucose iAUC at 120 and 180 min in comparison with a control scone containing 4.0 g of fiber [39]. Ultimately, Moazzami et al. found that there was no difference in PPG after the consumption of bread samples with diverse dietary fiber contents (ranging from 2.7 to 15.2 g) [63].

In conclusion, acute glycemic responses after the consumption of starchy foods rich in dietary fiber are not consistent among studies in individuals with overweight/obesity, and the results are mixed. In this population, more types of dietary fiber need to be tested, and more RCTs need to be conducted as well.

#### 3.3.2. Individuals with Different Health Conditions

In five out of the forty-one studies included in this systematic review, the authors included patients with various health statuses and overweight/obesity [35,51,52,53,66]. Patients with T2DM were assessed in three of the RCTS [35,51,52]. The study by Stringer et al. showed no differences in PPG or in glucose AUC after the consumption of rice (2.0 g fiber) or buckwheat crackers (3.2 g fiber) [52], while reductions in glucose iAUC, PPG, and peak glucose values were observed after the ingestion of pumpernickel rye bread (19.2 g fiber) compared to bread samples with lower fiber content in the study by Breen et al. [35]. The third study by Jenkins et al. revealed a decrease in the glycemic responses 180 min after the consumption of a high-fiber biscuit (11.6 g fiber) compared to white bread (2.5 g fiber), as well as a reduction in GI of 63% [51]. Moreover, Hartvigsen et al. investigated individuals with metabolic syndrome and found that bread meals enriched with β-glucan or arabinoxylan led to lower glucose iAUC, GI, and peak glucose levels in comparison with white bread [53]. Finally, in the study by Lappi et al. in healthy subjects with self-reported gastrointestinal symptoms after the ingestion of cereal foods, the consumption of bread samples fortified with soluble fiber in different ratios (arabinoxylan, fructan, and β-glucan) resulted in no differences in the glycemic responses or glucose iAUC between test meals, even though their fiber content ranged from 3.8 to 19.1 g [66].

In conclusion, the acute effects of dietary fiber in starchy foods on the glycemic responses are not well studied in individuals facing metabolic abnormalities. More studies are needed in this field to evaluate the impact of different dietary fiber types a few hours after the ingestion of starchy foods enriched with them. This is extremely important for patients with impaired glucose metabolism. The available data from the analysis cannot lead to solid conclusions.

### 3.4. Effects of Dietary Fiber on Insulinemic Responses

Twenty-five of the forty-one studies included in this systematic review measured the insulinemic responses after the consumption of starchy foods (alone or as part of a meal) differing in the amount and/or type of dietary fiber. These studies were conducted both in healthy volunteers [32,34,37,39,40,41,43,44,45,52,54,55,59,61,64,67,68,69,70,71,72] and in individuals with different health conditions [35,52,53,66].

#### 3.4.1. Healthy Individuals

##### Normal Weight

The majority of the studies evaluating the acute effects of dietary fiber in starchy foods on insulinemia were conducted in healthy volunteers with normal weight [32,34,37,40,41,43,44,45,54,55,58,59,61,67,68,70,71,72]. In eight studies, the effects of soluble dietary fiber alone were evaluated [37,44,45,54,55,58,59,72], while resistant starch was tested in five studies [40,41,61,67,68]. Three studies included foods with a combination of resistant starch and soluble and/or insoluble dietary fiber [32,70,71], and one RCT studied viscous cereal fiber [43], while in one trial, the type of fiber is not mentioned [34]. In the last study, Akhtar et al. found that the consumption of chapattis supplemented with either vegetable or bean powder resulted in the reduced amplitude of PPI compared to the control chapatti (100% wheat flour) [34]. However, the ingestion of viscous cereal fiber in whole-meal spaghetti (11.0 g fiber) did not result in different PPI in comparison with refined wheat spaghetti (3.0 g fiber), as the study by Costabile et al. showed [43]. Concerning resistant starch studies, four of them found a lower insulin iAUC after the ingestion of starchy foods (muffins, bread, and cookies) [40,41,61,67], while in the study by Johnson et al., chickpea bread (5.0 g fiber) increased the insulin iAUC and II compared to white bread (3.0 g fiber) [68]. Moreover, in the study by Stewart and Zimmer (2018), there were no differences in insulin Cmax between the fiber muffin (11.6 g fiber) and the control muffin (0.9 g fiber) [40], while in the study conducted by the same authors in 2017, they found a reduction in insulin Cmax of 23% after participants consumed a fiber cookie (24.13 g fiber) compared to the control cookie (0.55 g fiber) [41]. Additionally, the study by Belobrajdic et al. revealed lowered insulinemic responses to high-amylose wheat refined and whole-meal breads in comparison with the low-amylose one [61]. In contrast, the study by Poquette et al. revealed similar insulinemic responses to muffins with diverse resistant starch contents [67]. In addition to this are the studies examining the effects of soluble dietary fiber. Specifically, in three of the studies, a reduction in insulin iAUC was observed after the consumption of foods high in soluble fiber (buns, crispbreads, and flatbreads) compared to their control counterparts [37,58,59], and in three other studies, there were no differences in iAUC between the test meals [54,55,72], while in the study by Willis et al., in which different quantities of mixed soluble fiber were tested, it was found that a muffin containing 4.0 g of soluble fiber and 9.0 g of total dietary fiber resulted in elevated insulin iAUC compared with muffins containing 0, 8.0, and 12.0 g of soluble fiber [45]. Furthermore, in three trials, no differences in peak insulin were observed between the test meals (spaghetti, muffins, and biscuits) [45,55,72]. In terms of insulinemic responses, the results are also mixed. The RCTs by Juntunen et al. and by Johansson et al. reported improved insulinemic responses after the consumption of whole-kernel (12.8 g fiber) and β-glucan (17.1 g fiber) rye breads and unfermented (20.5 g fiber) and yeast-fermented (18.3 g fiber) wholegrain rye crispbreads, respectively [44,54]. Unfermented wholegrain rye crispbread also led to lower insulin secretion [54]. In contrast to these observations, two other RCTs showed no differences in insulinemic responses [58,67]. Ultimately, the consumption of starchy foods containing a mixture of soluble and insoluble dietary fiber led to reduced insulin iAUC in the study by Johnston et al. [70] but to higher insulin responses in the study by Hall et al., in which Australian lupin flour was used for bread preparation [71]. Ames et al. tested various tortillas differing in soluble and insoluble fiber and resistant starch and found that tortillas high in β-glucan (low insoluble fiber) reduced the insulin iAUC, but when the insoluble part was higher, there were no differences in iAUC [32].

In conclusion, it is not clear from the above results whether the consumption of starchy foods rich in dietary fiber has favorable effects on the acute insulinemic responses in healthy individuals with normal weight. However, there is consistency among reports showing favorable or no effects of soluble dietary fiber such as β-glucan and resistant starch, while the data concerning insoluble dietary fiber are limited. There is a great need for RCTs to resolve these issues.

##### Overweight and Obesity

Healthy individuals with overweight/obesity were assessed in three of the RCTs included [39,64,69]. In these studies, resistant starch was tested alone or in combination with soluble dietary fiber (guar gum and cellulose) [39,64], while the third study evaluated the effects of β-glucan [69], and the results are mixed. Firstly, in the last study, Najjar et al. found no differences in insulinemic responses, insulin sensitivity, or insulin iAUC, even though the test breads differed in total dietary fiber content, which ranged between 1.0 and 6.1 g [69]. Soluble dietary fiber and resistant starch co-inserted into a low-calorie muffin in the study by Quilez et al. resulted in lower insulinemic responses compared to white bread and a plain muffin [64]. Finally, Stewart et al. found that the consumption of scones containing 17.5 g of fiber (type-4 resistant starch) led to a lower venous iAUC after 120 and 180 min, as well as a lower insulin Cmax compared with the control scone (4.0 g fiber) [39].

In conclusion, the existence of resistant starch in two of the three studies could explain the ameliorated insulinemia in healthy individuals with overweight/obesity. However, the number of studies is not adequate to safely draw conclusions. More studies are needed in this population to evaluate not only resistant starch and soluble dietary fiber but also the short-term impact of insoluble fiber in starchy foods.

#### 3.4.2. Individuals with Different Health Conditions

Four out of the twenty-five studies assessed individuals with overweight/obesity and various health conditions [35,52,53,66]. Two studies were conducted in patients with T2DM [35,52]. In the study by Breen et al., in which different types of bread were tested in individuals with obesity, pumpernickel rye bread that contained 19.2 g of dietary fiber resulted in lower insulin iAUC_0–270_ and peak insulin compared to the other test breads, in which the fiber content ranged from 3.4 to 7.5 g [35]. Interestingly, both the white bread (3.4 g fiber) and wholegrain bread (7.2 g fiber) tested led to postprandial hyperinsulinemia 2 h after their consumption [35]. In contrast, the study by Stringer et al. showed no difference in PPI concentrations after the consumption of rice crackers (2.0 g fiber) and buckwheat crackers (3.2 g fiber) [52]. Furthermore, it was found by Hartvigsen et al. that the consumption of wheat bread with 24.4% arabinoxylan (11.2 g fiber) resulted in higher insulin iAUC_0–120_ compared to wheat bread with 13.3% oat β-glucan (13.4 g fiber), and that both of these breads led to higher insulin iAUC_0–120_ in comparison with rye bread with kernels (12.2 g fiber) in men and postmenopausal women with metabolic syndrome [53]. Finally, in the study by Lappi et al., in which a mixture of soluble dietary fiber in breads as part of a meal was tested, they found that the bread with the highest fiber content (19.1 gr, white bread fortified with native rye bran) resulted in a lower iAUC compared to other breads containing from 3.8 to 16.8 g of dietary fiber in healthy subjects with self-reported gastrointestinal symptoms after consuming cereal foods [66].

In conclusion, it is obvious from the above that more studies evaluating the acute effects of different types of dietary fiber on insulinemic responses in patients with metabolic abnormalities are needed. In particular, it is crucial to assess whether there is a difference in acute insulinemic responses of individuals with prediabetes or T2DM after the intake of starchy foods high in soluble or insoluble fiber or resistant starch.

## 4. Discussion

This systematic review did not reveal favorable acute effects of dietary fiber incorporated in different starchy products on glycemic and insulinemic responses among healthy individuals and participants with various health conditions. In contrast, it instead confirmed the inconsistency that dominates in the literature. Specifically, it was shown that soluble dietary fiber has no clear effect on glycemia in individuals with normal weight, while resistant starch may be more effective in flattening postprandial glycemic responses. Regarding insulinemic responses, both soluble fiber and resistant starch produced mixed results, with either favorable or no effects. Data on insoluble dietary fiber and glucose metabolism are scarce in this population. The same mixed results for glycemia can be seen in healthy volunteers with overweight/obesity, while resistant starch seems to improve postprandial insulin responses. Finally, there are not enough studies examining the acute effects of dietary fiber in starchy foods on glucose metabolism and insulin secretion in individuals with glucose abnormalities to draw conclusions.

Starchy products such as bread, spaghetti, cookies, etc., are the most commonly consumed foods in modern societies and lead to the elevation of blood glucose levels [30]. Their wholegrain versions are high in dietary fiber, as the whole part of the grain is used for their production [75]. The predominant opinion is that the consumption of foods high in dietary fiber, either alone or as part of a meal, leads to improved glycemic and insulinemic responses [11]. This has been proved in long-term studies in patients with T2DM [28,76]. However, this favorable effect occurs after modifying not only the diet (toward high fiber consumption) but also the total quality of life, e.g., by increasing physical activity and reducing alcohol consumption. Consequently, it is not obvious whether dietary fiber alone can result in improved postprandial glucose and insulin values.

Carbohydrates in products are the main nutrients affecting blood glucose levels. When these products are low in dietary fiber and have a high GI or GL, they are easily digestible and rapidly absorbable, leading to higher blood glucose excursions. Chronic hyperglycemia can lead to the dysfunction of pancreatic β-cells, thus lowering insulin secretion. Moreover, when there is an over-abundance of energy, i.e., high GL, body tissues such as skeletal muscle, adipose tissue, and the liver become resistant to insulin action [77]. Dietary habits that continually expose tissues and cells to sustained post-meal hyperglycemia can impair first-phase insulin secretion and insulin action in insulin-sensitive tissues, increasing the risk for the development of insulin resistance and T2DM [34]. In the short term, a lower insulin response prevents hypoglycemia and inappropriate increases in non-esterified fatty acids (NEFA) and anti-insulin hormone responses, often seen during the late postprandial period after the intake of refined carbohydrates [24]. The regular consumption of diets with low postprandial insulin responses, e.g., rye-pasta diets, may also benefit individuals with impaired first-phase insulin secretion by allowing the β-cell function to recover, leading to improved insulin secretion in the long term [59]. Specifically, insulin is secreted from the pancreas in a biphasic manner in response to a square wave increase in systemic glucose concentrations. The first phase of insulin release consists of a brief spike, followed by the second phase, which reaches a peak at about 60 min or more depending on the carbohydrate, protein, and lipid contents of the meal [24]. It is widely thought that the diminution of first-phase insulin secretion is the earliest detectable defect of pancreatic β-cell function in individuals at high risk for T2DM; this defect largely represents β-cell exhaustion after years of compensation for antecedent insulin resistance. Τhe first phase of insulin secretion is totally absent in individuals with very high blood glucose concentrations [78]. In subjects with T2DM, the restoration of the first phase of insulin secretion after a mixed meal improved postprandial hyperglycemia and suppressed endogenous lipolysis, resulting in the decrease of plasma NEFA levels [79].

The inconsistency in results can be attributed to specific aspects of the different studies, such as the amount of dietary fiber used, dietary fiber properties (fermentability, gel-forming, and molecular weight and size), the preparation method and food matrix (rye or whole-wheat bread or pasta), and the nutrient composition of the food [43]. To date, only a few mechanisms of action have been described as regards the effects of dietary fiber on glucose homeostasis [80]. As was analyzed earlier, dietary fiber, depending on its type, can delay the rate of gastric emptying. This results in the decreased absorption of macronutrients such as fat and glucose [81]. The delay in gastric emptying and intestinal glucose absorption after a meal plays an important role in the regulation of postprandial hyperglycemia [24]. This is the reason why dietary fiber, specifically soluble β-glucan, has obtained the health claim from the FDA for lowering blood cholesterol levels in a quantity equivalent to 3.0 g/day [82]. The second health claim for dietary fiber states that, along with the lower consumption of fats (<30% of total energy intake), the increased consumption of dietary fiber from fruits, vegetables, and wholegrain products may reduce the risk of some types of cancer [83].

Dietary fiber could act by deranging some of the carbohydrate content that would normally be absorbed in the small intestine or could move carbohydrates to lower parts of the intestinal tract, where less of an effect on insulin secretion would be observed [84]. The slower carbohydrate digestion process may lead to a slower elevation and/or decreased peak in blood glucose levels [85,86]. In people with type 1 diabetes mellitus, the delay in intestinal glucose absorption with first- and second-generation α-glucosidase inhibitors after a meal can decrease postprandial glucose excursions and insulin requirements [87,88]. However, some studies measuring gastric emptying did not show that the consumption of high-fiber starchy products led to delayed emptying [44,50,89,90]. This may be attributed either to differences in the total carbohydrate content [50] or to small differences in fiber amounts between the test foods [44,50]. Soluble dietary fiber has been linked to reduced glucose fluctuations due to enhanced digesta viscosity after the ingestion of meals containing viscous dietary fiber [91,92,93]. It is believed that guar gum has the greatest impact on postprandial glycemia due to its highest viscosity resulting in the inhibition of digestive enzymes [94]. However, gastrointestinal secretions and dilution, and acidification and re-neutralization, may impact the rheological properties of these polysaccharides in vivo [91]. Moreover, β-glucan from barley or oats is the most studied soluble dietary fiber in the literature [25,94]. In long-term studies, this type of fiber has been associated with improved glycemic control [91,95], although the results are mixed concerning its acute effects. In 2011, EFSA reported that individuals who wish to reduce PPG should consume 4 g of β-glucans from oats or barley for every 30 g of available carbohydrates per meal [96]. This can probably be explained by other parameters influencing its actions. Two of these factors may be the degree of processing and the molecular weight of β-glucan [94]. Fiber with greater viscosity, higher molecular weight, and less processing may lead to a reduction in peak glycemic responses [47,94]. It is also likely that β-glucan, due to its high viscosity in the gastrointestinal tract, not only reduces postprandial glucose responses but also decreases starch digestion by a-amylase [38].

A more viscous chyme slows nutrient digestion and absorption at the beginning of the small intestine. As a result, nutrients reach the distal ileum and stimulate mucosal L-cells to release glucagon-like peptide (GLP-1) into the bloodstream [84,94]. This peptide stimulates pancreatic β-cells, enhancing insulin production and sensitivity, and lowers glucagon secretion from α-cells, inhibiting liver glucose production [91]. Another incretin hormone that is involved in postprandial glucose metabolism is glucose-dependent insulinotropic polypeptide (GIP), which collaborates with GLP-1 to stimulate postprandial insulin secretion [97]. In contrast to soluble dietary fiber, the consumption of insoluble dietary fiber accelerates GIP and insulin responses, acting through peripheral mechanisms [98,99,100]. In the study by Boers et al., the main cause of reduced postprandial insulin responses to the test flatbreads with a fiber-and-flour mix was probably slower intestinal glucose absorption, leading to the decreased stimulation of incretin secretion, notably GIP, the release of which is directly related to the site and rate of glucose absorption [59]. Moreover, in the study by Juntunen et al., glycemic responses did not differ between rye products (bread and pasta) and white bread (reference food); however, insulinemic, GLP-1, and GIP responses were lower after the consumption of rye bread and pasta, apart from GLP-1 responses to rye bread containing an oat β-glucan concentrate [44]. However, in another study by Belobrajdic et al., while the consumption of high-amylose bread resulted in 30% lower GIP, GLP-1, and iAUC compared to low-amylose bread, there were no differences in incretin secretion between the whole-meal and refined flour breads [61]. Differences in GLP-1 concentration were not observed after the consumption of whole-meal spaghetti (11.0 g of fiber) compared to refined wheat spaghetti (3.0 g of fiber) [43].

Another possible mechanism that seems to be the link between dietary fiber intake and the reduction in T2DM risk is fermentability [101]. In particular, dietary fiber such as resistant starch, which ends up undigested in the colon, can be fermented by the bacterial flora and hence result in the production of short-chain fatty acids (SFA) such as acetate, propionate, and butyrate [102,103,104]. These may help in the improvement of glucose tolerance and the reduction in PPG responses [105,106]. It is known that NEFA circulating in the blood can inhibit glucose metabolism through the blockage of glucose transporter type 4 (GLUT-4) [107] and may increase insulin secretion from pancreatic β-cells due to higher glycolytic flux and mitochondrial respiration [108]. As a result, the release of SFA from the gut microbiota decreases serum NEFA levels induced by insulin resistance and may help improve blood glucose responses through competition in insulin-sensitive tissues, e.g., adipose and muscle, leading to increased glucose uptake [107,109]. Furthermore, SFA act on intestinal endocrine cells and/or in neurons of the enteric nervous system to change gastrointestinal motility and secretion [110]. These molecules also behave as signaling molecules, activating G protein-coupled receptors (GPCRs), especially GPR41 and GPR43 on the brush border membrane, and thus, they stimulate the release of GLP-1 [111].

In the majority of meals, dietary fiber is accompanied by other macronutrients. Specifically, the protein and fat contents of foods may impact glycemic and insulinemic responses [112]. It has been proved that the co-ingestion of large amounts of fat with a carbohydrate meal has glucose-lowering effects even in healthy subjects, without a concomitant reduction in plasma insulin levels [113]. Additionally, protein intake seems to stimulate insulin secretion, thus resulting in glucose uptake and its reduction in the bloodstream [85,86,114]. However, the amount of protein content that is capable of inducing higher insulin responses is not yet established [114]. The test foods in the studies included in this systematic review differed to some extent in the fat, total carbohydrate, and protein contents, in addition to the type and/or quantity of dietary fiber. This may be an important reason for the differentiation between the glycemic and insulinemic responses observed.

Finally, meeting the recommendation for daily dietary fiber intake is challenging. This reflects an average consumption of 28 g/day in the context of a 2000 kcal diet [13]. Some of the studies included in the final analysis tested products with fiber levels approaching this number [32,41,54,57,72], and the acute results on glucose metabolism are consistently mixed. Specifically, the consumption of unfermented wholegrain rye crispbread containing 20.2 g of mixed soluble dietary fiber and resistant starch resulted in same glycemic responses and lower insulinemic responses and insulin secretion compared to lower doses of fiber and different fermentation statuses [54]. In the studies by Papakonstantinou et al. and Soong et al., the ingestion of 21.4 and 21.1 g of dietary fiber (both soluble) from starchy products, respectively, did not result in differences in glycemic and/or insulinemic responses compared to the other test foods [57,72], while only the consumption of a barley muffin led to lower peak glucose values [57]. In the study by Stewart and Zimmer (2017) in normal-weight healthy volunteers, the consumption of a cookie containing 24.13 g of fiber, mainly as resistant starch, led to reduced responses in comparison with the control cookie (0.55 g fiber) [41]. In contrast to this observation, the ingestion of a tortilla made with bran flour and high insoluble and medium β-glucan (26.87 g fiber) contents resulted in similar glycemic and insulinemic responses compared to a tortilla made with wholegrain flour and medium insoluble and β-glucan contents (14.28 g fiber) [32].

It is clear from the above that there are some limitations in the studies that do not allow the extraction of generalized conclusions. The results of this systematic review prove that more studies need to be conducted on the acute effects of dietary fiber on glycemia and insulinemia. Factors that are known to affect glycemia, e.g., the protein and/or fat content of foods, need to be considered in future research. These factors should be kept constant between test foods/meals. Starchy foods should differ only in the type, i.e., soluble, insoluble, and resistant starch, and amount of dietary fiber. Along with these factors, another parameter that should be taken into consideration is the type of starchy food that is used each time. Moreover, these effects should be further investigated in individuals with overweight or obesity, as well as in those with glucose abnormalities.

## 5. Conclusions

In summary, although the long-term positive effects of dietary fiber intake on glucose metabolism have been well studied and established, the results of studies measuring acute postprandial glycemia and insulinemia are not consistent with these findings. The majority of the studies were conducted in healthy volunteers with normal weight, and the results are mixed. Soluble dietary fiber does not seem to be superior in lowering glycemic and insulinemic responses at an acute level, as supported by long-term studies; in contrast, resistant starch may acutely improve glycemia in healthy individuals with normal weight and insulinemia in volunteers with overweight/obesity. Ultimately, more studies are needed to prove whether the consumption of a high-fiber carbohydrate-containing product per se can result in blunted glycemic and insulinemic responses in individuals with impaired glucose metabolism and insulin resistance and whether the improved indices observed in interventional studies are attributed to the overall improvement of the quality of life, e.g., by increasing physical activity or adopting a specific dietary pattern, and not to the dietary fiber consumption alone. All other factors affecting these responses should be eliminated to see the exact impact of each dietary fiber type separately on glycemia and insulinemia.

## Figures and Tables

**Table 1:** Characteristics of the studies included in the analysis.

Reference	Country	Study Design and Duration (min)	Health Status Sample Size Age (Years) Sex BMI (kg/m^2^)	Type of Dietary Fiber	Test Meals (Macronutrients and Dietary Fiber Analysis)	Glycemic Responses	Insulinemic Responses
Juntunen et al., 2002 [44]	Finland	RX 180	Healthy subjects 20 28.0 ± 1.0 10Μ:10F 22.9 ± 0.7	Oat β-glucan (soluble DF)	(50 g AVCHO) Wheat WB as reference food (TDF: 3.1 g, 0.9 g soluble, 2.0 g pentosan, 0.2 g β-glucan/Pr: 8.4 g/L: 3.0 g) Whole-kernel rye bread (WKRB) (60% whole rye kernels and 40% rye flour) (TDF: 12.8 g, 3.8 g soluble, 8.7 g pentosan, 1.3 g β-glucan/Pr: 7.4 g/L: 2.6 g) β-Glucan rye bread (RB) (20% oat β-glucan and 80% rye flour) (TDF: 17.1 g, 6.8 g soluble, 10.3 g, pentosan, 5.4 g β-glucan/Pr: 10.5 g/L: 2.4 g) + 40 g cucumber + 0.3 L non-energy-containing orange drink Whole-meal pasta (WMP) (dark durum wheat) (TDF: 5.6 g, 1.3 g soluble, NM g pentosan, NA g β-glucan/Pr: 12.1 g/L: 4.7 g) + 19 g crushed tomatoes, 0.3 L non-energy-containing orange drink	β-Glucan RB vs. wheat WB: ↑ GR at 120 min WMP vs. wheat WB: ↑ GR at 120, 150, 180 min WMP vs. wheat WB: ↓ maximal GR	WKRB vs. wheat WB: ↓ InsR at 30, 45, 60, 90, 120, 150 min β-Glucan RB vs. wheat WB: ↓ InsR at 45, 60, 120, 150, 180 min WKRB, β-glucan RB, WMP vs. wheat WB: ↓ maximal InsR WKRB, WMP vs. wheat WB: Smaller PPI areas above fasting levels
Hall et al., 2005 [71]	Australia	RX, single-blind 120	Healthy subjects 11 31.6 ± 1.8 9M:2F 24.7 ± 0.8	Soluble and insoluble DF	(50 g AVCHO) Breakfast with WB (TDF: 2.8 g/Pr: 9.2 g/L: 6.4 g) Breakfast with Australian Sweet Lupin Flour Bread (ASLF) (TDF: 4.9 g/Pr: 12.8 g/L: 7.3 g) + 6 g low-fat margarine, 20 g low-joule apricot spread, a cup of decaffeinated tea with 30 g skim milk	ASLF (GI = 52) vs. WB (GI = 100): ↓ GI	ASLF vs. WB: ↑ Ins index
Johnson et al., 2005 [68]	Australia	RX, single-blind 120	Healthy subjects 12 32.0 ± 2.0 10M:2F 24.7 ± 0.8	RS	(50 g AVCHO) WB (TDF: 3.0 g/Pr: 9.0 g/L: 6.0 g) Chickpea bread (CHB) (TDF: 5.0 g/Pr: 11.0 g/L: 7.0 g) Extruded chickpea bread (EXB) (TDF: 6.0 g/Pr: 11.0 g/L: 7.0 g) + margarine, jam, milk, and tea	CHB vs. WB: ↓ GR at 90 min Trend toward ↓ iAUC EXB vs. WB: ↓ GR at 120 min ND in GI between test meals	WB, EXB vs. CHB: peak at 30 vs. 45 min CHB vs. WB, EXB: ↑ InsR at 60 min CHB vs. WB: ↑ Ins iAUC ↑ Ins index
Quilez et al., 2007 [64]	Spain	RX 120	Healthy subjects 14 33.1 ± 7.8 7M:7M 25.8 ± 2.9 Group 1 (normal): 20 to 24.9 Group 2 (overweight): 25 to 29.9	Carboxy-methyl cellulose and guar gum (soluble DF) High-amylose corn starch (RS)	(50 g AVCHO) Bread as reference food (TDF: 2.7%/CHO: 56.5%/Pr: 8.8%/L: 0.7%) Plain muffin (PM) (TDF: 1.5%/CHO: 47.9%/Pr: 4.8%/L: 21.2%) Low-calorie muffin (LCM) (TDF: 6.3%/CHO: 49.2%/Pr: 5.0%/L: 10.3%)	Bread vs. LCM: Differences in GR Bread, LCM vs. PM: ND in GR Overweight vs. normal: ↑ GR with bread and LCM ND in GR with PMs LCM vs. bread: ↓ 51.8% GR	Bread, PM vs. LCM: ↑ InsR PM, bread vs. LCM: ↑ 69.7% and ↑ 63.3% InsR
Jenkins et al., 2008 [51]	Canada	RX single-blind Trial 1: 90 Trial 2: 180	Trial 1: Healthy participants 10 28.0 ± 2.6 4M:6F 24.3 ± 0.8 Trial 2: Participants with T2DM 9 68.0 ± 3.8 3M:6F 28.8 ± 1.2	PGX: Glucomannan (soluble DF) Xanthan (soluble DF)	(50 g AVCHO) C Biscuit (CB) (TDF: 1.7 g/CHO: 51.8 g/Pr: 4.5 g/L: 10.2 g) Biscuit with 10 g of fiber blend (FB) (TDF: 11.6 g/CHO: 63.1 g/Pr: 4.3 g/L: 10.9 g) WB (TDF: 2.5 g/CHO: 52.6 g/Pr: 8.3 g/L: 0.4 g) WB with 12 g of margarine (WBM) (TDF: 2.5 g/CHO: 52.6 g/Pr: 8.4 g/L: 10.0 g)	Trial 1: FB (GI = 26) vs. CB (GI = 101), WB, WBM (GI = 108): ↓ 74% GI FB vs. WB: ↓ GR at 30, 45, 60, 90 min ND in GR between CB, WB, and WBM Trial 2: FB (GI = 37) vs. CB (GI = 94), WB, WBM (GI = 103): ↓ 63% GI FB vs. WB: ↓ GR at 30, 60, 90, 120, 150, 180 min ND in GR between CB, WB and WBM	NA
Hlebowicz et al., 2009 [50]	Sweden	RX 90	Healthy subjects 10 26.0 ± 1.0 3M:7F 24.1 ± 0.8	Rye β-glucan and arabino-xylan (soluble DF)	(50 g AVCHO per meal) WB (TDF: 0.0 g/AVCHO: 52.0 g/Pr: 13.5 g/L: 0.0 g) Rye whole-meal bread (TDF: 3.75 g/AVCHO: 62.7 g/Pr: 12.75 g/L: 4.5 g) + ham, 300 mL FUN Light fruit drink	ND in GR and iAUC between test meals	NA
Marinangeli et al., 2009 [42]	Canada	RX, single-blind 150	Healthy individuals 19 22–67 7M:12F 21–42	Soluble and insoluble DF	(50 g AVCHO) WB as reference food Boiled yellow peas (BYP) as reference food Banana bread with whole yellow pea flour (WYPF) (TDF: 8.1 g, 3.0 g soluble, 5.1 g insoluble/CHO: 52.0 g/Pr: 9.3 g/L: 15.2 g) Banana bread with WWF (TDF: 7.1 g, 3.1 g soluble, 4.0 g insoluble/CHO: 51.7 g/Pr: 7.8 g/L: 16.1 g) Biscotti with WYPF (TDF: 10.1 g, 3.3 g soluble, 6.4 g insoluble/CHO: 51.7 g/Pr: 12.2 g/L: 13.3 g) Biscotti with WWF (TDF: 8.2 g, 3.3 g soluble, 4.4 g insoluble/CHO: 53.2 g/Pr: 9.5 g/L: 13.3 g) Spaghetti with 30% WYPF and 70% white wheat durum (TDF: 8.1 g, 3.4 g soluble, 4.8 g insoluble/CHO: 51.1 g/Pr: 7.4 g/L: 1.6 g) Spaghetti with 100% whole-wheat durum (TDF: 6.6 g, NA g soluble or insoluble/CHO: 51.1 g/Pr: 9.9 g/L: 1.2 g)	WYPF biscotti and WYPF banana bread vs. WB: ↓ 61.9% and 55.1% iAUC WYPF spaghetti vs. BYP: ↑ 43.1% iAUC ND in iAUC between WYPF and WWF spaghetti WYPF vs. WWF biscotti: ↓ 29.2% iAUC WYPF biscotti (GI = 45.4), WYPF banana bread (GI = 50.3) vs. WYPF spaghetti (GI = 93.3): ↓ GI WYPF biscotti (GI = 45.4) vs. WWF biscotti (GI = 63.9): ↓ GI	NA
Najjar et al., 2009 [69]	Canada	RX, single-blind 180	Overweight or obese males 11 59.0 ± 2.41 11M 30.8 ± 0.95	β-Glucan (soluble DF)	(50 g AVCHO) WB (TDF: 1.5 g/Pr: 9.7 g/L: 4.2 g) Whole-wheat bread (WWB) (TDF: 6.3 g/Pr: 16.0 g/L: 6.1 g) Sourdough bread (SB) (TDF: 1.0 g/Pr: 9.8 g/L: 4.7 g) Whole-wheat barley bread (WWBB) (TDF: 6.1 g/Pr: 15.1 g/L: 6.1 g)	SB vs. WB, WWB: ↓ GR WWBB vs. WWB: ↓ GR SB vs. WWB: ↓ Glu AUC	ND in InsR, Ins AUC, and Ins sensitivity index between test meals
Thondre and Henry, 2009 [47]	United Kingdom	RX, single-blind 120	Healthy subjects 8 38.0 ± 11.0 3M:5F 23.2 ± 3.5	Barley β-glucan (soluble DF)	(50 g AVCHO) Glu as reference food 5 chapattis (CH) containing 0, 2, 4, 6, and 8 g of β-glucan (+WWF) CH0 (TDF: 9.1 g, 0 g β-glucan/CHO: 59.1 g/Pr: 10.9 g/L: 1.5 g) CH2 (TDF: 11.3 g, 2 g β-glucan/CHO: 62.5 g/Pr: 11.9 g/L: 1.64 g) CH4 (TDF: 13.1 g, 4 g β-glucan/CHO: 63.2 g/Pr: 12.4 g/L: 1.7 g) CH6 (TDF: 15.2 g, 6 g β-glucan/CHO: 65.2 g/Pr: 13.1 g/L: 1.7 g) CH8 (TDF: 17.2 g, 6 g β-glucan/CHO: 67.0 g/Pr: 13.8 g/L: 1.8 g)	CH2, CH4, CH6, CH8 vs. Glu: ↓ GR ND in GR between CH0 and CH2 CH4: ↓ GR at 45 min CH8: ↓ GR at 45 and 60 min CH4 and CH8 vs. CH0: ↓ GI CH0, CH2, CH4, CH6, CH8 vs. Glu: ↓ Glu iAUC_0–120_ CH4, CH8 vs. CH0: ↓ Glu iAUC_0–120_ CH0, CH2, CH4, CH6, CH8 vs. Glu: ↓ ΔGlu at 15, 30, 45 min CH4 and CH8 vs. CH0: ↓ 43% and 47% GI Glu: peak time at 30 min CH0, CH2, CH6: peak time at 45 min CH4: peak time at 60 min CH8: peak time at 30 min and maintained until 60 min	NA
Kristensen et al., 2010 [36]	Denmark	RX open-labeled 180	Young healthy adults 16 24.1 ± 3.8 6M:10F 21.7 ± 2.2	Arabinoxylans (soluble DF)	4 isocaloric meals (50 g AVCHO per meal) WB (TDF: 3.9 g/CHO: 45% of E/Pr: 20.5% of E/L: 34.4% of E) Wholegrain WB (WWB) (TDF: 11.7 g/CHO: 51.7% of E/Pr: 19.8% of E/L: 28.4% of E) Refined wheat pasta (RWP) (TDF: 2.2 g/CHO: 44.1% of E/Pr: 21.3% of E/L: 34.5% of E) Wholegrain pasta (WWP) (TDF: 5.0 g/CHO: 48.2% of E/Pr: 21% of E/L: 30.9% of E) + cheese	ND in GR between bread meals or pasta meals at any time point WB vs. RWP: ↑ GR at 30, 45, 60, 90 min ↑ Glu AUC WWB vs. WWP: ↑ GR at 45 and 60 min ↑ Glu AUC RWP vs. WB: ↓ GI	NA
Vuksan et al., 2010 [56]	Canada	RX, double-blind 120	Healthy subjects 11 30.0 ± 3.6 6M:7F 22.2 ± 1.3	NM	(50 g AVCHO) WB (TDF: 2.1 g/CHO: 52.1 g/Pr: 9.4 g/L: 0.7 g) Low-Salba-dose bread (LSB) (TDF: 4.9 g/CHO: 54.9 g/Pr: 11.11 g/L: 3.1 g) Intermediate-Salba-dose bread (ISB) (TDF: 8.1 g/CHO: 58.1 g/Pr: 13.1 g/L: 5.7 g) High-Salba-dose bread (HSB) (TDF: 11.7 g/CHO: 61.7 g/Pr: 15.3 g/L: 8.7 g)	LSB, ISB, HSB vs. WB: ↓ 41%, 28%, and 21% iAUC HSD vs. WB: ↓ GR at 30, 45, 60 min ISB vs. WB: ↓ GR at 60 min LSB vs. WB: ↓ GR at 45 min	NA
Chillo et al., 2011 [33]	United Kingdom	RX 120	Healthy subjects 9 35.0 ± 11.6 3M:6F 21.7 ± 4.1	GlucaGel (GG): 79.4% low-molecular-weight β-glucan (soluble DF) Barley Balance (BB): 26.5% high-molecular-weight β-glucan (soluble DF), 10% other DF	(50 g AVCHO) Glu as reference food Spaghetti samples: 0GG/0BB (WDS) 2GG (2%GG + WDS) 4GG (4%GG + WDS) 6GG (6%GG + WDS) 8GG (8%GG + WDS) 10GG (10%GG + WDS) 2BB (2%BB + WDS) 4BB (4%BB + WDS) 6BB (6%BB + WDS) 8BB (8%BB + WDS) 10BB (10%BB + WDS)	All GG spaghetti vs. Glu: ↓ GR at 15, 30, 45 min 4GG, 6GG, 8GG, 10GG vs. Glu: ↓ GR at 60 min Similar GR at 90 and 120 min Glu and all GG spaghetti: Peak time at 30 min All BB spaghetti vs. Glu: ↓ GR at 15, 30, 45 min 4BB, 6BB, 8BB, 10BB vs. Glu: ↓ GR at 60 min BB: peak time at 45 min All GG spaghetti vs. Glu: ↓ iAUC (mean ↓ 47%) 6GG and 10GG vs. Glu: ↓ 32.6% and 29.5% iAUC All BB spaghetti vs. Glu: ↓ iAUC (mean ↓ 60%) 10BB vs. 0BB: ↓ 51.6% iAUC ↑ %BB → ↓ GI 10BB vs. 0BB: ↓ 55% GI	NA
Thondre and Henry, 2011 [48]	United Kingdom	RX, single-blind 120	Healthy subjects 10 35.0 ± 7.5 6M:4F 23.1 ± 2.4	GG^TM^ as source of β-glucan (75% β-glucan) (soluble DF)	(50 g AVCHO) Glu as reference food Chapatti with 0% β-glucan (TDF: 9.1 g/CHO: 59.0 g/Pr: 10.9 g/L: 1.5 g) Chapatti with 4% β-glucan (TDF: 12.6 g, 4 g β-glucan/CHO: 60.0 g/Pr: 10.5 g/L: 1.5 g) Chapatti with 8% β-glucan (TDF: 14.3 g, 8 g β-glucan/CHO: 61.0 g/Pr: 10.1 g/L: 1.5 g)	ND in iAUC between test meals 0, 4, 8% chapattis vs. Glu: ↓ AUCs ND in AUCs between chapatti samples 4% (GI = 55), 8% (GI = 52) vs. 0% (GI = 58) chapatti: ↓ GI	NA
Willis et al., 2011 [45]	USA	RX, double-blind 180	Healthy subjects 20 26.0 ± 7.0 10M:10F 24.0 ± 2.0	Mixed soluble DF: pectin, barley β-glucan, guar gum, pea fiber, and citrus fiber	4 muffins (MUF) containing 0, 4, 8, and 12 g of mixed fiber MUFF0 (TDF: <1.0 g, ΝA g soluble or insoluble/CHO: 74.0 g/Pr: 11.0 g/L: 20.0 g) MUFF4 (TDF: 6.0 g, 3.0 g soluble, 3.0 g insoluble/CHO: 81.0 g/Pr: 12.0 g/L: 13.0 g) MUFF8 (TDF: 9.0 g, 4.0 g soluble, 5.0 g insoluble/CHO: 89.0 g/Pr: 12.0 g/L: 10.0 g) MUFF12 (TDF: 13.0 g, 6.0 g soluble, 7.0 g insoluble/CHO: 93.0 g/Pr: 13.0 g/L: 13.0 g)	MUFF0 vs. MUFF4, MUFF8, MUFF12: ↓ Glu AUC ↓ mean change in peak Glu from baseline ↑ dose → ↑ Glu AUC MUFF12 vs. MUFF4, MUFF8: ↓ mean change in peak Glu from baseline	MUFF4 vs. MUFF0, MUFF8, MUFF12: ↑ Ins AUC ND in mean change in peak Ins from baseline between test meals
Aldughpassi et al., 2012 [38]	Canada	RX 120	Healthy participants 10 40.6 ± 2.7 4M:6F 27.6 ± 1.2	β-Glucan (soluble)	(50 g AVCHO) WB as reference food Wholegrain and white pearled test meals (different barley cultivars) AC Parkhill (high amylose, low β-glucan) Celebrity (high amylose, medium β-glucan) CDC Fibar (low amylose, high β-glucan)	All 6 test meals vs. WB: ↓ GR CDC Fibar (wholegrain) vs. AC Parkhill (wholegrain): ↓ GR and GI CDC Fibar (pearled) vs. CDC Fibar (wholegrain): ↑ GI	NA
Lappi et al., 2013 [66]	Finland	RX 240	Healthy subjects with self-reported gastrointestinal symptoms after ingestion of cereal foods, particularly rye bread 15 57.0 6M:9F 26.0	Arabinoxylan Fructan β-Glucan (soluble DF)	(50 g AVCHO) WB (TDF: 3.8 g, 1.3 g total arabinoxylan, 0.8 g soluble arabinoxylan, 0.4 g fructan, 0.2 g β-glucan/Pr: 9.6 g/L: 6.1 g) White Wheat Rye Bread (WWR) (TDF: 16.4 g, 5.3 g total arabinoxylan, 1.7 g soluble arabinoxylan, 2.4 g fructan, 1.7 g β-glucan/Pr: 9.2 g/L: 1.0 g) WB fortified with bioprocessed rye bran (BRB) (TDF: 16.8 g, 8.3 g total arabinoxylan, 3.8 g soluble arabinoxylan, 1.2 g fructan, 0.8 g β-glucan/Pr: 15.8 g/L: 10.1 g) WB fortified with native rye bran (WWRB) (TDF: 19.1 g, 7.6 g total arabinoxylan, 1.5 g soluble arabinoxylan, 2.0 g fructan, 2.3 g β-glucan/Pr: 14.5 g/L: 9.7 g) + 40 g cucumber, 20 g milk-free margarine, and 3 dl water or 1.75 dl filtered coffee or black tea	ND in GR and iAUC between test meals	WWRB vs. WB, BRB, WWRB: ↓ InsR at 60 min ↓ Ins iAUC
Breen et al., 2013 [35]	Ireland	RX 270	Subjects with T2DM and obesity 10 53.9 ± 5.5 6M:4F 35.1 ± 7.5	NM	(50 g AVCHO) WB (TDF: 3.4 g/Pr: 10.8 g/L: 1.17 g) Whole-meal Soda Bread (WSB) (TDF: 7.4 g/Pr: 9.6 g/L: 2.2 g) WGB (TDF: 7.5 g/Pr: 12.9 g/L: 2.9 g) Pumpernickel rye Bread (PRB) (TDF: 19.2 g/Pr: 10.2 g/L: 3.9 g)	PRB vs. WGB: ↓ mean iAUC PRB vs. WB, WSB, WGB: ↓ peak Glu PRB vs. WB: ↓ 2-h PPG WSB, WGB, WB: 2 h postprandial hyperglycemia (>140 mg/dL) PRB, WSB vs. WB, WGB: ↓ peak time (55.5, 58.5 vs. 75.0, 72.0 min)	PRB vs. WB, WGB: ↓ iAUC ↓ peak Ins WSB, WGB, WB: 2 h postprandial hyperinsulinemia
Kristensen et al., 2013 [37]	Denmark	RX double-blind 420	Young men 17 27.2 ± 2.2 18M 25.4 ± 2.2	Flaxseed DF (70–80% water-soluble DF)	4 iso-caloric meals 2 buns with cheese, butter, ham, and different flaxseed fractions C (TDF: 7 g/CHO: 147 g/Pr: 44 g/L: 49 g) Whole flaxseed (WF) (TDF: 12 g/CHO: 147 g/Pr: 44 g/L: 49 g) Low-dose mucilage (LM) (TDF: 12 g/CHO: 147 g/Pr: 44 g/L: 50 g) High-dose mucilage (HM) (TDF: 17 g/CHO: 147 g/Pr: 44 g/L: 49 g)	ND in PPG and AUC_0–180_ between test meals	HM vs. C: ↓ InsR at 30 min HM vs. WF: ↓ InsR at 30 and 180 min LM vs. C, WF: ↓ AUC_0–180_ HM vs. C, WF: ↓ AUC_0–180_
Stringer et al., 2013 [52]	Canada	RX, single-blind 180	Trial 1: Healthy subjects with HbA1c < 6% 11 37.3 ± 16.3 6M:6F 23.5 ± 3.4 Trial 2: Subjects with well-controlled T2DM with HbA1c < 7.5% 12 60.8 ± 6.7 5M:7F 32.4 ± 6.6	NM	(50 g AVCHO) Rice crackers (TDF: 2.0 g/CHO: 51.8 g/Pr: 4.0 g/L: 8.1 g) Buckwheat crackers (TDF: 3.2 g/CHO: 53.1 g/Pr: 10.7 g/L: 10.6 g)	ND in PPG and AUC_0–180_ between test meals	NA
Hartvigsen et al., 2014 [53]	Denmark	RX 270	Men and postmenopausal women with MetS 15 62.8 ± 4.2 7M:8F 31.1 ± 3.2	Arabinoxylan (soluble DF) β-glucan (soluble DF) (PromOat)	(50 g AVCHO) WB (TDF: 2.9 g/Pr: 9.0 g/L: 2.3 g) Wheat bread with 13.3% of oat β-glucan (BG) (TDF: 13.4 g/Pr: 9.8 g/L: 2.5 g) Wheat bread with 24.4% of wheat arabinoxylan (AX) (TDF: 11.2 g/Pr: 19.4 g/L: 2.6 g) Rye bread with kernels (RK) (TDF: 12.2 g/Pr: 7.3 g/L: 7.3 g)	BG (GI = 84%), RK (GI = 77%) vs. WB (GI = 100%): ↓ GI BG, RK vs. WB: ↓ Glu iAUC_0–120_ AX, BG, RK vs. WB: ↓ peak Glu	AX, WB, BG vs. RK: ↑ Ins iAUC_0–120_ AX vs. BG: ↑ Ins iAUC_0–120_
Moazzami et al., 2014 [63]	Sweden	RX 180	Healthy postmenopausal women 19 61.0 ± 4.8 19F 26.0 ± 2.5	Soluble and insoluble DF	(50 g AVCHO) Sourdough containing both yeast and lactobacilli was used in all RBs Refined wheat bread (RWB) as reference bread (TDF: 2.7 g, 1.5 g insoluble, 1.2 g soluble/Pr: 9.0 g/L: 5.2 g) Refined rye bread (RRB) (TDF: 6.1 g, 3.1 g insoluble DF, 3.0 g soluble/Pr: 4.9 g/L: 3.4 g) Whole-meal rye bread (WRB) (TDF: 15.2 g, 10.9 g insoluble and 4.3 g soluble/Pr: 11.1 g/L: 7.8 g) + 40 g cucumber and 3 dl noncaloric orange drink	ND in PPG responses between breads at 30, 45, 60, 180 min WRB vs. RWB: ↑ GR at 90 min ND in GR between RRB and the other bread samples	NA
Poquette et al., 2014 [67]	USA	RX 180	Healthy subjects 10 25.1 ± 4.0 10M 24.2 ± 2.8	RS	(50 g AVCHO) Whole-Wheat Flour Muffin (WWF) (Pr: 7.8%/L: 16.0%) Wholegrain Sorghum Muffin (WGS) (Pr: 5.2%/L: 18.5%/↑ RS content)	WGS vs. WGW: ↓ GR at 45, 60, 75, 90, 120 min ↓ 26% Glu iAUC_0–120_ ND in GR at 180 min between test meals	WGS vs. WGW: ↓ InsR at 15, 30,45, 60, 75, 90 min ↓ 55% Ins iAUC_0–120_ ND in InsR at 180 min between test meals
Ames et al., 2015 [32]	Canada	RX, double-blind 180	Healthy subjects 12 27.0 7M:5F 23.8	β-Glucan (soluble DF) Insoluble DF RS	(50 g AVCHO) Glu as reference food Barley tortilla made from: Straight-grade flour (SGF)—low β-glucan/low soluble DF (TDF: 10.29 g, 7.55 g insoluble, 4.5 g β-glucan, 0.45 g RS/Pr: 13.67 g) Wholegrain flour (WGF)—medium β-glucan/low insoluble DF (TDF: 14.28 g, 7.43 g insoluble, 7.77 g β-glucan, 0.42 g RS/Pr: 13.28 g) Bran flour with β-glucan/low insoluble DF (BF-BG) (TDF: 18.03 g, 7.47 g insoluble, 11.55 g β-glucan, 0.85 g RS/Pr: 14.71 g) Bran flour with high insoluble DF/medium β-glucan (BF-IDF) (TDF: 26.87 g, 19.64 g insoluble, 8.56 g β-glucan, 0.68 g RS/Pr: 21.50 g) High-amylose dusted flour fractions (HA-DFF)—medium β-glucan/low insoluble DF (TDF: 14.14 g, 8.29 g insoluble, 6.27 g β-glucan, 1.41 g RS/Pr: 12.08 g)	BF-BG (GI = 22.7) vs. SGF (GI = 51.8), WGF (GI = 57.3): ↓ 56–60% GI ND in GI between WGF and HA-DFF (GI = 39.2) and between WGF and BF-IDF (GI = 40.9) BF-BG vs. SGF: ↓ 61% Glu iAUC BF-BG vs. SGF, WGF: ↓ 3.9–5.1 times change from baseline at 30 min ND in Glu iAUC or %change from baseline at 30 min between WGF and HA-DFF or between WGF and BF-IDF	HA-DFF, BF-BG vs. SGF: Returned to baseline at 120 vs. 180 min BF-BG vs. WGF: ↓ 39% Ins iAUC WGF vs. SGF: ↓ 33% Ins iAUC SGF vs. WGF, BF-BG: ↑ 64–176% change from baseline at 30 min ND in Ins iAUC between WGF and BF-IDF BF-IDF vs. WGF: ↑ Ins %change from baseline at 30 min ND in iAUC between WGF and HA-DFF
Johansson et al., 2015 [54]	Sweden	RX 230	Healthy subjects 23 60.1 ± 12.1 7M:16F 23.8 ± 3.4	Arabinoxylan Arabinogalactan β-Glucan Cellulose and RS Fructan Klason lignin	Unfermented wholegrain rye crispbread (uRCB) (TDF: 20.5 g–8.8 g arabinoxylan, 0.1 g arabinogalactan, 2.5 g β-glucan, 2.7 g cellulose and RS, 4.0 g fructan, 1.3 g Klason lignin) Yeast-fermented wholegrain rye crispbread (RCB) (TDF: 18.3 g–8.6 g arabinoxylan, 0.2 g arabinogalactan, 2.1 g β-glucan, 2.5 g cellulose and RS, 2.6 g fructan, 1.3 g Klason lignin) Yeast-fermented refined wheat crispbread (WCB) (TDF: 6.0 g–2.5 g arabinoxylan, 0.2 g arabinogalactan, 0.3 g β-glucan, 1.4 g cellulose and RS, 0.4 g fructan, 0.5 g Klason lignin) + margarine and cheese, a glass of orange juice, and a cup of coffee or tea	ND in PPG between test meals	uRCB, RCB vs. WCB: ↓ InsR at 65 and 95 min uRCB vs. RCB, WCB: ↓ 13% and 17% Ins secretion, ↓ 12% and 21% InsR RCB vs. WCB: ND in AUC_0–230_ RCB vs. WCB: ↓ 10% InsR
Soong et al., 2015 [57]	Singapore	RX, non-blind 120	Healthy subjects 12 26.2 ± 5.3 4M:8F 20.2 ± 1.7	Oat and barley β-glucan (soluble DF)	(50 g AVCHO) Glu as reference food Wheat Muffins (WM) (TDF: 3.7 g/CHO: 91.0 g/Pr: 14.1 g/L: 1.5 g) Rice Muffins (RM) (TDF: 3.2 g/CHO: 99.2 g/Pr: 6.4 g/L: 1.6 g) Corn Muffins (CM) (TDF: 17.7 g/CHO: 79.5 g/Pr: 8.8 g/L: 4.5 g) Oat Muffins (OM) (TDF: 12.8 g/CHO: 70.4 g/Pr: 22.4 g/L:9.6 g) Barley Muffins (BM) (TDF: 21.4 g/CHO: 76.8 g/Pr: 12.8 g/L: 0 g)	WM, CM, BM vs. RM, OM: peak Glu at 30 vs. 45 min WM, RM, CM vs. BM: ↑ peak Glu WM (GI = 74), RM (GI = 79), CM (GI = 74) vs. BM (GI = 55): ↓ 120 min period GR OM (GI = 53): ↓ iAUC at 45 min OM: rapid ↓ GR at 45 min WM, RM, CM, BM: gradual ↓ GR WM, CM, OM, BM vs. RM: Glu above baseline at 120 min	NA
Robert et al., 2016 [46]	Malaysia	RX 120	Healthy individuals 10 21–48 5M:5F 20.0–30.2	Galacto-mannan from fenugreek seed (soluble, viscous DF)	(50 g AVCHO) Glu as reference food Bun C (CB) (TDF: 3.0 g/Pr: 9.4 g/L: 5.0 g) 10% Fenugreek Bun (FB) (TDF: 12.0 g/Pr: 15.0 g/L: 5.5 g) Flatbread C (CF) (TDF: 3.0 g/Pr: 9.0 g/L: 1.4 g) 10% Fenugreek Flatbread (FF) (TDF: 6.0 g/Pr: 10.4 g/L: 3.0 g)	FB (GI = 51) vs. CB (GI = 82): ↓ 39.2% Glu AUC (GR) ↓ 38% GI FF (GI = 43) vs. CF (GI = 63): ↓ 30.4% Glu AUC (GR) ↓ 32% GI	NA
Mohan et al., 2016 [49]	India	RX 120	Healthy volunteers Study 1 (2013): 25 27.9 ± 0.9 13M:12F 22.3 ± 0.5 Study 2 (2014): 15 26.7 ± 1.0 7M:8F 20.6 ± 0.4	RS	(50 g AVCHO) Glu as reference food WR (Per 100 g of uncooked rice—TDF: 1.58 g, 0.6 g RS/AVCHO: 77.1 g/Pr: 9.4 g/L: 0.8 g) High-fiber WR (HFWR) (Per 100 g of uncooked rice—TDF: 8.0 g, 3.9 g RS/AVCHO: 75.1 g/Pr: 8.0 g/L: 0.3 g)	Mean values from the 2 studies HFWR (GI = 61.3) vs. WR (GI = 79.2): ↓ 23% GI, and iAUC	NA
Stamataki et al., 2016 [55]	Greece	RX 180	Healthy subjects 11 22.4 ± 1.6 6M:5F 23.2 ± 2.8	Inulin (soluble DF)	(50 g AVCHO) Glu as reference food Biscuit samples: oat flakes (40%), whole-wheat flour (60%) Oat biscuits (OB) (TDF: 5.1 g/Pr: 6.8 gr/L: 15.3 g) Oat biscuits with 4% inulin (OBIN) (TDF: 5.4 g, 3.3 g inulin/Pr: 8.0 g/L: 14.0 g)	OB vs. OBIN: ↓ peak Glu at 45 min OB, OBIN vs. GS: ↓ iAUC ND in iAUC between OB and OBIN OBIN (GI = 45.68) vs. OB (GI = 32.82): ↑ GI	OBIN vs. OB: ↑ Ins at 45, 60 min ND in iAUC between test meals OBIN vs. OB: Peak Ins at 45 min vs. 30 min ND in peak Ins between test meals
Boers et al., 2017 [59]	India	RX, double-blind 180	Healthy South-Asian subjects 50 29.16 ± 0.71 30M:26F 20.77 ± 0.20	β-Glucan (soluble DF)	100% wheat-flour-based flatbread as C (TDF: 8.0 g/AVCHO: 65.0 g) Flatbread samples 2% guar gum (80 g HFF + 15 g CPF + 3 g BF) (TDF: 16.0 g/AVCHO: 56.0 g) 3% guar gum (77 g HFF + 15 g CPF + 5 g BF) (TDF: 17.0 g/AVCHO: 54.0 g) 4% guar gum (81 g HFF + 15 g CPF) (TDF: 18.0 g/AVCHO: 53.0 g)	3, 4% guar gum vs. C: ↓ iAUC_0–120_	2, 3, 4% guar gum vs. C: ↓ Ins iAUC_0–120_
Khan et al., 2017 [65]	Australia	RX 120	Healthy subjects 20 21.5 ± 1.15 10M:10F 17.97 ± 3.08	NM	(50 g AVCHO) C cookies (CC) made with plain wheat flour (TDF: 0.58 g/Pr: 7.88 g/L: 1.02 g) Cookies containing 3% bay leaf powder (B3) (TDF: 0.99 g/Pr: 8.07 g/L: 1.13 g) Cookies containing 6% bay leaf powder (B6) (TDF: 1.4 g/Pr: 8.27 g/L: 1.24 g)	B6 vs. CC: ↓ GR at 30 and 45 min ND in iAUC_0–120_ between test meals	NA
Stewart and Zimmer, 2017 [41]	USA	RX, double-blind 120	Healthy subjects 28 42.8 ± 18.5 14M:14F 24.7 ± 3.3	VERSAFIBE^TM^ 1490 RS type 4	C cookie (CC) (TDF: 0.55 g/AVCHO: 36.28 g/Pr: 5.36 g/L: 3.99 g) Fiber cookie (FC) (TDF: 24.13 g/AVCHO: 12.71 g/Pr: 4.92 g/L: 3.92 g)	FC vs. CC: ↓ intravenous Glu at 45 min ↓ capillary Glu at 15, 30, 45, 60, 90, 120 min ↓ 44% intravenous and ↓ 48% capillary Glu iAUC_0–120_ ↓ 8% intravenous and ↓ 9% capillary Cmax_0–120_	FC vs. CC: ↓ intravenous Ins at 45, 60, 90, 120 min ↓ 46% intravenous Ins iAUC_0–120_ and ↓ 23% Cmax_0–120_
Zamaratskaia et al., 2017 [58]	Sweden	RX, single-blind 250	Healthy subjects 23 30.0 ± 11.0 13M:11F 23.0 ± 5.0	Arabinoxylan Arabinogalactan β-Glucan Cellulose and RS Fructan Klason lignin	Refined wheat crispbread as C Yeast-fermented refined wheat crispbread (TDF: 6.0–2.5% arabinoxylan, 0.2% arabinogalactan, 0.3% β-glucan, 1.4% cellulose, and RS, 0.4% fructan, 0.5% Klason lignin) (TDF: 2.9 g/CHO: 35.0 g/Pr: 6.5 g/L: 4.1 g) Unfermented rye crispbread (TDF: 20.5–8.8% arabinoxylan, 0.1% arabinogalactan, 2.5% β-glucan, 2.7% cellulose and resistant starch, 4.0% fructan, 1.3% Klason lignin) (TDF: 11.7 g/CHO: 35.4 g/Pr: 6.0 g/L: 0.9 g) Sourdough-fermented rye crispbread (TDF: 17.5–8.2% arabinoxylan, 0.2% arabinogalactan, 2.1% β-glucan, 2.7% cellulose, and resistant starch, 1.7% fructan, 1.5% Klason lignin) (TDF: 9.5 g/CHO: 36.2 g/Pr: 5.1 g/L: 1.1 g) + coffee/tea (150 mL), margarine, cheese, and juice (150 mL)	ND in PPG responses between test meals	Unfermented rye vs. sourdough-fermented rye, C: ↓ Ins AUC_0–230_ Sourdough-fermented rye vs. C: ND in Ins AUC_0–230_ ND in PPI responses between test meals
Stewart and Zimmer, 2018 [40]	USA	RX double-blind 120	Healthy adults 28 41.1 ± 17.2 14M:14F 24.5 ± 3.4	VERSAFIBE^TM^ 2470 Resistant Starch (RS) type 4 with 70% DF	C Muffin Top (CMT) (TDF: 0.9 g/CHO: 29.0 g/AVCHO: 28.1/Pr: 4.0 g/L: 4.3 g) Fiber Muffin Top (FMT) (TDF: 11.6 g/CHO: 29.0 g/AVCHO: 17.4/Pr: 4.0 g/L: 4.3 g)	FMT vs. CMT: ↓ venous blood Glu at 15 and 30 min ↓ 33% venous Glu iAUC_0–120_ ↓ 8% venous Glu Cmax _0–120_ ↓ capillary blood Glu at 30 min	FMT vs. CMT: ↓ venus Ins at 30, 45, 60 min ↓ Ins iAUC_0–120_ ND in venous Ins Cmax _0–120_
Akhtar et al., 2019 [34]	Pakistan	RX 120	Normoglycemic healthy young adults 24 M: 21.1 ± 1.2/F: 23.8 ± 2.6 12M:12F M: 22.5 ± 1.7/F: 21.0 ± 1.7	NM	(50 g AVCHO) Glu as reference food All-purpose wheat flour chapatti (APFC)-100% wheat flour (TDF: 3.60 g/Pr: 5.05 g/L: 0.81 g) Vegetable-powder-supplemented chapatti (VPSC)-20% vegetable powder (TDF: 6.72 g/Pr: 8.62 g/L: 0.90 g) Bean-powder-supplemented chapatti (BPSC)-25% bean powder (TDF: 5.33 g/Pr: 8.92 g/L: 1.14 g) + fried egg cooked with sunflower oil	BPSC (GI = 44) and VPSC (GI = 46) vs. APFC (GI = 82): ↓ 46% and 44% GI BPSC and VPSC vs. APFC: ↓ GR at 15, 30, 45 min ↓ 44% and 49% iAUC_0–120_	VPSC and BPSC vs. APFC: ↓ InsR at 60 min ↓ amplitude of PPI
Belobrajdic et al., 2019 [61]	Australia	RX 180	Healthy subjects 19 30.0 ± 3.0 5M:15F 23.0 ± 0.7	RS (fermentable DF)	Glu as reference food Per 100 g bread: High-amylose wheat refined bread (HAW-R) (TDF: 5.5 g, 4.7 g RS/Pr: 13.1 g/L: 2.8 g) High-amylose wheat whole-meal bread (HAW-W) (TDF: 10.4 g, 3.2 g RS/Pr: 15.2 g/L: 3.6 g) Low-amylose wheat refined bread (LAW-R) (TDF: 3.3 g, 0.4 g RS/Pr: 10.8 g/L: 3.4 g) Low-amylose wheat whole-meal bread (LAW-W) (TDF: 8.2 g, 0.3 g RS/Pr: 12.1 g/L: 3.7 g)	HAW vs. LAW breads: ↓ 39% Glu iAUC ↓ 33% Glu Cmax	HAW vs. LAW breads: ↓ 24% Ins iAUC HAW-W vs. LAW-R: ↓ InsR at 60 and 120 min HAW-R vs. LAW-R: ↓ InsR at 120 min
Matsuoka et al., 2020 [60]	Japan	RX, double-blind 120	Healthy subjects 23 22.8 ± 1.4 7M:16F 21.0 ± 2.6	β-Glucan (soluble DF)	(50 g AVCHO) Wheat flour bread (WFB) (TDF: 1.8 g, 0.19 g β-glucan/Pr: 8.5 g/L: 4.9 g) Barley flour bread (BFB) (TDF: 3.0 g, 2.5 g β-glucan/Pr: 16.8 g/L: 6.3 g) +180 mL lactose-free milk	BFB vs. WFB: ↓ peak Glu	NA
Yoshimoto et al., 2020 [62]	Japan	RX, double-blind 120	Healthy subjects 12 37.8 ± 9.5 8M:4F 22.9 ± 3.5	Legumes (insoluble DF)	WB as reference food Legume-based noodle samples: Dehulled yellow pea noodles (YP) (TDF: 5.3 g/%RAG: 8.34/CHO: 23.1 g/Pr: 10.1 g/L: 1.0 g) Unshelled yellow pea noodles (YP-U) (TDF: 7.8 g/%RAG: 8.20/CHO: 20.0 g/Pr: 8.7 g/L: 1.0 g)	YP, YP-U vs. WΒ: ↓ GR at 45, 60, 90 min YP vs. YP-U: ↓ GR at 45 min ND in iAUC between YP and YP-U	NA
Papakonstantinou et al., 2022 [72]	Greece	RX, single-blind 120	Healthy individuals 14 25.0 ± 1.0 4M:10F 23.0 ± 1.0	Soluble DF	(50 g AVCHO) Glu as reference food WB as reference food Spaghetti made with hard WDS flour (S) (TDF: 1.8 g/CHO: 72.0 g/Pr: 12.0 g/L: 1.5 g) Wholegrain spaghetti made with wholegrain hard wheat flour (WS) (TDF: 7.0 g/CHO: 67.6 g/Pr: 12.8 g/L: 2.1 g) Spaghetti high in soluble fiber and low in CHO made with hard WDS flour, rice bran, oat fibers, and flaxseed flour (HFLowCS) (TDF: 21.1 g/CHO: 47.4 g/Pr: 14.9 g/L: 4.6 g)	S, WS, HFLowCS vs. Glu, WB: ↓ GR at 15, 30, 45, 60 min with ND between them ↓ peak Glu S, WS vs. Glu: ↓ GR at 90 min WS vs. Glu: ↑ GR at 120 min S vs. WB: ↓ GR at 30 and 120 min S, WS, HFLowCS: ↓ GR at 45, 60, 90 min S vs. WS, HFLowCS: ↓ peak Glu S, WS, HFLowCS vs. Glu: ↓ Glu iAUC_0–120_ ND in Glu iAUC_0–120_ between the three types of spaghetti S vs. WB: ↓ Glu iAUC_0–120_	ND in salivary Ins between test meals and Glu or WB ND in iAUC_0–120_, peak Ins, and time to peak between test meals

Abbreviations: Randomized controlled crossover trial = RX; BMI = body mass index; M = males; F = females; DF = dietary fiber; Glu = glucose; WDS = wheat durum semolina; GR = glycemic responses; ↓ = lower; ↑ = higher; GI = glycemic index; GL = glycemic load; AUC = area under the curve; iAUC = incremental area under the curve; AVCHO = available carbohydrates; NM = not mentioned; TDF = total dietary fiber; CHO = carbohydrates; Pr = protein; L = lipids; InsR = insulin responses; PPG = postprandial glucose; PPI = postprandial insulin; T2DM = type 2 diabetes mellitus; WB = white bread; WGB = wholegrain bread; Ins = insulin; C = control; ND = no difference; WWF = whole-wheat flour; ΝA = not applicable; E = energy content; PGX = PolyGlycopleX; WR = white rice; RAG = rapidly available glucose; Cmax = maximal concentration; HFF = high-fiber flour; CPF = chickpea flour; GG = guar gum; BF = barley flour; MetS = metabolic syndrome.

## Data Availability

Not applicable.
